# A Chronic Obstructive Pulmonary Disease Self-Management Intervention for Improving Patient-Reported Outcomes in Primary Care in Greece

**DOI:** 10.3390/medicina60030377

**Published:** 2024-02-23

**Authors:** Filothei Tsaousi, Izolde Bouloukaki, Antonios Christodoulakis, Despo Ierodiakonou, Nikos Tzanakis, Ioanna Tsiligianni

**Affiliations:** 1 Department of Social Medicine, School of Medicine, University of Crete, 71500 Heraklion, Greece; filothey@gmail.com (F.T.); izolthi@gmail.com (I.B.); christodoulakisa@icloud.com (A.C.); 2 Department of Nursing, School of Health Sciences, Hellenic Mediterranean University, 71410 Heraklion, Greece; 3 Department of Primary Care and Population Health, Medical School, University of Nicosia, 2417 Nicosia, Cyprus; ierodiakonou.d@unic.ac.cy; 4 Department of Thoracic Medicine, University Hospital of Heraklion, School of Medicine, University of Crete, 71003 Heraklion, Greece; tzanakisn@uoc.gr

**Keywords:** COPD, self-management, primary healthcare

## Abstract

*Background and Objectives*: Self-management programs are essential for increasing COPD patient participation and autonomy in making appropriate decisions about their chronic condition. The present study aimed to assess the impact of COPD self-management interventions on quality of life, functional status, patient education, depression, and anxiety in primary care. *Materials and Methods*: We conducted a randomized controlled trial recruiting patients with COPD (GOLD A and B) from four primary care centers in Crete, Greece, with one intervention group (*n* = 40) receiving self-management educational support and one control group (*n* = 80) receiving usual care. To measure quality of life, functional status, patient education, depression, and anxiety, we used patient-reported outcome measures (PROMs) at baseline and 6 months post-intervention, including the Short-Form Health survey (SF-12), Clinical COPD Questionnaire (CCQ), mMRC, Beck Anxiety Inventory (BAI), Beck Depression Inventory, Health Education Impact Questionnaire (HeiQ), and Health Literacy Questionnaire (HLQ). *Results*: At the end of the 6-month intervention, most PROMs improved significantly in the intervention group (*p* < 0.05) but did not show significant changes in the control group. The greatest improvements at follow-up compared to baseline measurements were observed for dyspnea (mMRC—38.6%), anxiety (BAI—35%), depression (BDI—20.2%), COPD health status (CCQ—34.1%), and the actively managing my health subscale of HLQ (23.5%). *Conclusions*: Our results suggest that a self-management intervention could be an effective strategy for improving PROMs in primary care. Although more research is needed to identify the long-term effects of such interventional programs, policymakers could implement similar programs to improve the overall health of these patients.

## 1. Introduction

Chronic obstructive pulmonary disease (COPD) is a common lung disease characterized by progressive airflow limitation that is not fully reversible [1]. According to the World Health Organization, COPD will be the third leading cause of morbidity and mortality worldwide by 2030, causing significant disability, poor quality of life (QoL), and high utilization of healthcare resources [2,3]. COPD, even with appropriate treatment, is difficult to manage due to frequent exacerbations that may necessitate hospitalization [4,5]. Nevertheless, the symptoms and clinical course of the disease can be modified by effectively implementing health behaviors, such as exercise, smoking cessation, stress management, breathing exercises, adherence to medications, and exacerbation management [6,7,8].

Considering that COPD is a progressive disorder and symptoms may often change, self-management programs are essential to increase patient involvement and autonomy in making appropriate decisions about their own chronic condition [9,10,11]. Although various definitions exist, self-management programs in general are supportive interventions provided by healthcare staff, peers, or non-professionals with the goal of increasing patients’ skills and confidence in managing their disease [12]. These programs seem to help patients improve their knowledge about their disease and reduce exacerbations, admission rates, and overall healthcare costs [11,13]. Furthermore, they may assist patients with COPD in responding to changing symptoms and potentially improve their quality of life [9,10,11].

However, previous studies were cautiously optimistic about the potential of self-management programs to improve the quality of life and decrease emergency visits, and the results have been inconclusive [14]. Furthermore, trials have primarily recruited people from secondary and tertiary care, and excluded those with mild disease [15]. This is of great importance, since most patients with COPD receive care in primary care settings rather than hospitals [11,16]. The available evidence on self-management interventions for COPD patients in primary care has produced conflicting results regarding the impact on health-related quality of life [9,17,18]. Moreover, the impact of self-management on hospitalization rates, quality of life, self-efficacy, and healthcare utilization remains inconclusive [15]. Nevertheless, positive effects were observed in adherence to medication, physical activities, and smoking cessation [15]. To the best of our knowledge, no previous study has examined the effects of self-management programs on patients with COPD in Greece.

Taking into account the aforementioned, it is crucial to conduct further research on the use of self-management programs in primary healthcare practice. Therefore, the aim of our study was to evaluate the implementation and clinical effectiveness of a COPD self-management intervention compared to usual care focusing on the following outcomes: functional and mental status, patient health literacy, and quality of life.

## 2. Materials and Methods

### 2.1. Study Design and Population

We conducted a prospective randomized controlled parallel-group trial of standard versus self-management educational intervention support for patients with COPD. A hundred and twenty consecutive patients with COPD were recruited from four primary care centers in Heraklion city in Crete, Greece, between April 2020 and August 2020 during a regular consultation meeting. We included patients based on the following criteria: (1) aged ≥ 18 years, (2) had previously been diagnosed with COPD by a physician (Group GOLD A and B according to mMRC and number of exacerbations), (3) stable on their medications (no treatment modifications) in the past three months, (4) able to speak, read, and/or comprehend Greek. The exclusion criteria were: refusal to participate, concurrent oncological diseases, severe cognitive impairment, and neurological or psychiatric disabilities.

### 2.2. Randomization and Blinding

**Figure 1:**
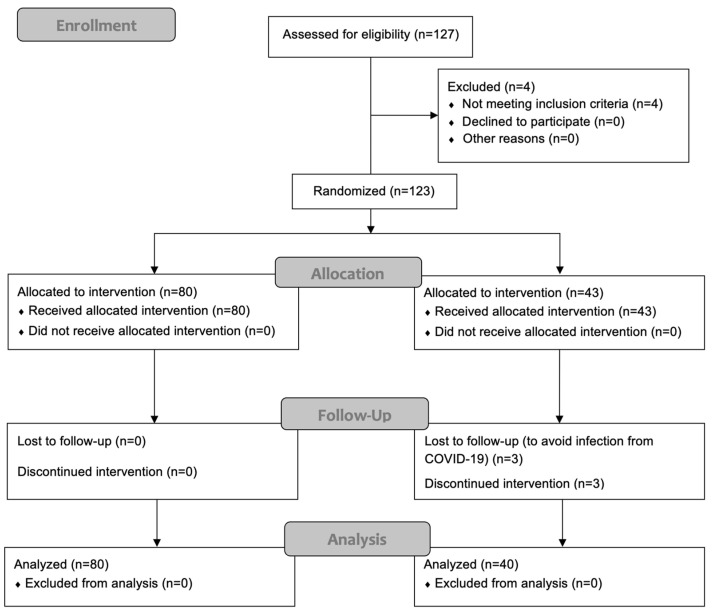
Overview of recruitment, allocation, and randomization procedure.

Randomization was performed using a computer-generated table of random numbers prepared by a biostatistician and maintained by the staff at the clinical center. Individuals were randomized (1:2) to the control group (*n* = 80), receiving usual follow-up care, or the intervention group (*n* = 40), with follow-up care based on an additional self-management intervention program (Figure 1). Patients were blinded to the group to which they were allocated and were monitored for a duration of 6 months. 

### 2.3. Usual Care

Taking into account the aforementioned, it is crucial to further explore the use of self-management programs in primary healthcare practice. Therefore, the aim of our study was to assess the implementation and clinical effectiveness of a COPD self-management intervention compared to usual care focusing on the following outcomes: functional and mental status, patient health literacy, and quality of life.

Patients who were assigned to receive usual care were advised to maintain their regular medical appointments and their check-ups/reviews, and contact the health coach if they had any questions. The participants in this group received no additional advice, information, or recommendations. Study-related contact occurred through telephone calls due to the COVID-19 pandemic, which collected healthcare utilization data (see Supplementary Materials) at baseline and at the follow-up visit at 6 months.

### 2.4. Self-Management Intervention

The self-management program included a “training book”, which was created for the study by the Department of Social Medicine, Faculty of Medicine, University of Crete, Greece, and monthly sessions with a coach; all coaches received training in motivational interviewing methods. The implementation process involved five steps, which included monthly sessions for five months. Additionally, there was a coach present during the sessions and follow-up phone calls after each session concluded. The educational sessions were held by at least one coach, and covered (a) Pathophysiology of COPD, (b) Risk Factors, (c) Symptom Recognition, (d) Staging of the Disease and Recognition of the Stage to Which the Individual Belongs, (e) Exacerbation Recognition Training, (f) Exacerbation Prevention Training, (g) Training with Breathing Strengthening Exercises and Muscle Strengthening Exercises, and (h) Proper Use of Inhalers. The sessions aimed to motivate and involve patients by incorporating a range of educational methods, such as sharing information, group discussions, practical training, and assignments. Detailed information about the self-management program and the data collection process can be found in the Supplementary Materials.

### 2.5. Study Outcomes and Measurements

Patients’ data were collected prior to the start of the sessions (baseline assessment) and 6 months after, at the end of the study (follow-up assessment). All participants completed a self-reported questionnaire that included demographic characteristics of the participants, such as age, sex, marital status, educational level, employment status, area of residence, body mass index (BMI), and smoking habits. Additional information was retrieved from patients’ medical records.

The primary outcome was to assess the potential effect of the self-management intervention on quality of life, functional status, patient education, depression, and anxiety using patient-reported outcome measures (PROMs), namely: Short-Form Health survey (SF-12), for assessing quality of life [19,20], Clinical COPD Questionnaire (CCQ) [21,22] and mMRC for clinical evaluation of COPD [23], Beck Anxiety Inventory (BAI), for assessing anxiety [24,25], Beck Depression Inventory, 2nd edition (BDI) for evaluating depression [24,26,27], Health Education Impact Questionnaire (HeiQ), for evaluating the training intervention and self-management [28,29,30], and Health Literacy Questionnaire (HLQ), for assessing health literacy [31,32]. For all questionnaires we used translated validated versions and obtained permission to use the questionnaires that required permission.

#### 2.5.1. Short-Form Health Survey (SF-12)

The SF-12 questionnaire, which consists of 12 items selected from the SF-36 [20], is used as a shorter alternative to the SF-36 to assess the impact of health on an individual’s everyday life. It is one of the most widely used tools for evaluating self-reported health-related quality of life. It covers the same health domains as the SF-36 and provides the physical and mental health summaries (PCS-12, MCS-12) as two summary scores with substantially fewer questions, making it a more practical research tool. Higher scores on SF-12 indicate better physical and mental health functioning.

#### 2.5.2. Clinical COPD Questionnaire (CCQ)

The Clinical COPD Questionnaire (CCQ) is a ten-item, self-administered tool. It was designed primarily to assess health status in primary care settings, but it is also useful in clinical trials for measuring response to intervention. The CCQ is divided into three sections: symptoms, functional state, and mental state. The questions apply to the previous week and are graded on a seven-point scale ranging from zero to six. The CCQ total score is calculated as the mean of the sum of all items, with a higher value indicating lower health status [21]. In our study, CCQ score was expressed both as the CCQ total score and as mean scores of the domains. The minimal difference in CCQ score considered to be of clinical importance is 0.4 [33].

#### 2.5.3. Modified Medical Research Council (mMRC) Dyspnea

The British scale of the Medical Research Council (MRC) was developed to assist physicians establish clinical grades of breathlessness (five grades) for their patients with COPD. Today, a modified version of this scale is used (mMRC), which, on a scale of 0 to 4, assesses the degree of disability caused by shortness of breath in daily activities [23]. Furthermore, the mMRC’s assessment of dyspnea is now used to classify symptomatic burden of COPD according to the Global Initiative for Chronic Obstructive Lung Disease (GOLD) recommendations and it provides useful information about COPD-induced disability [34]. Higher scores indicate greater severity of breathlessness.

#### 2.5.4. Beck’s Depression Inventory (BDI)

Beck’s Depression Inventory (BDI) was used to evaluate depression levels. It is scored by summing the ratings for each of the 21 items. Each item is rated on a 4-point scale from 0 to 3, with total scores ranging from 0 to 63. Higher scores indicate increased severity of depression. BDI total scores of 0–13 indicate minimal signs of depression; 14–19, mild depression; 20–28, moderate depression; and 29–63, severe depression [35].

#### 2.5.5. Beck Anxiety Inventory (BAI)

The Beck Anxiety Inventory (BAI) is also a 21-item checklist that measures cognitive, affective, and physiological symptoms of anxiety [25,36]. The application and scoring are quick, and the patient rates the severity of symptoms over the last seven days on a scale of 0 (not at all) to 3 (severely). The responses are summed to produce a single score ranging from 0 to 63. The total score of 0–7 indicates minimal anxiety, 8–15 mild anxiety, 16–25 moderate anxiety, and 26–63 severe anxiety [37].

#### 2.5.6. Health Education Impact Questionnaire (HeiQ)

The HeiQ is a self-report measure that comprises 40 items scored on a Likert scale ranging from one (strongly disagree) to four (strongly agree) [28,29,30]. This questionnaire evaluates patient education and self-management interventions among individuals with a wide range of chronic conditions, including COPD. It is divided into eight dimensions, each of which represents a different set of self-management capacities and skills: 1. Positive and active engagement in life, 2. Health directed activities, 3. Skill and technique acquisition, 4. Constructive attitudes and approaches, 5. Self-monitoring and insight, 6. Health service navigation, 7. Social integration and support, and 8. Emotional distress. Each dimension is assessed by four to six questions, for a total of forty questions. The responses are marked on a 4-point scale.

#### 2.5.7. Health Literacy Questionnaire (HLQ)

The HLQ is a 44-item measure assessing nine distinct domains of health literacy in order to capture the lived experiences of people attempting to comprehend, access, and use health information and services [32]. The nine scales are: (1) Feeling understood and supported by healthcare providers; (2) Having sufficient information to manage my health; (3) Actively managing my health; (4) Social support for health; (5) Appraisal of health information; (6) Ability to actively engage with healthcare providers; (7) Navigating the healthcare system; (8) Ability to find good health information; (9) Understand health information enough to know what to do. The response options for domains 1 to 5 are on a scale of 1 to 4 (strongly disagree to strongly agree). Domains 6 to 9 have a scale ranging from 1 to 5 (cannot do to very easy).

### 2.6. Statistical Analysis

Data analysis was conducted utilizing IBM SPSS version 25.0. In cases where variables are normally distributed, the results are presented as mean ± standard deviation (SD), while, for non-normally distributed variables, the median (25th–75th percentile) is reported. The presentation of qualitative variables involves expressing them as absolute numbers along with their corresponding percentages. Baseline comparisons between the two groups were assessed with Chi-squared or Fisher’s exact tests for categorical variables, and with Student’s T-test for continuous variables or a Mann–Whitney U test if not normally distributed. Reliability of the questionnaires included internal consistency assessment using the Cronbach’s alpha coefficient. Changes in the values of the patient outcomes in each group were compared over time using paired *t* tests (for normally distributed data) or Wilcoxon Signed test (for non-normally distributed data) for dependent samples, while generalized linear model for repeated measures was used to assess the effectiveness of the intervention on all outcomes. For the analysis, important prognostic factors such as age, place of residence, age at diagnosis of COPD, and the number of exacerbations in 2019 were adjusted for using analysis of covariance. A significance level of 0.05 was set as acceptable.

## 3. Results

### 3.1. Differences in Clinical Characteristics between the Two Groups

The sociodemographic and health status characteristics of the participants are described in Table 1. Most of the participants were men (62%), 66 years old or older (54%), married or in a relationship (73%). There was a significantly higher proportion of patients in the intervention group who lived in urban areas compared to the control group. Differences in other evaluated characteristics, including age, other comorbidities, and smoking status, remained relatively insignificant between the two groups (all *p* > 0.05). Both groups had a similar age of COPD diagnosis (53 vs. 57, *p* = 0.05); however, a higher median number of exacerbations was noted in the intervention group compared to the control group (1.7 vs. 1.0, *p* = 0.005). Some of the PROMs were also significantly (*p* < 0.05) different between the groups at baseline: BAI (17 vs. 7), BDI (17 vs. 10), CCQ (2.1 vs. 1.5), and mMRC (2 vs. 1) (Table 2). Concerning the questionnaires’ reliability, all Cronbach’s alphas indicated acceptable internal consistency and reliability (Cronbach’s α-coefficients ≥ 0.70). 

### 3.2. Follow Up

**Figure 2:**
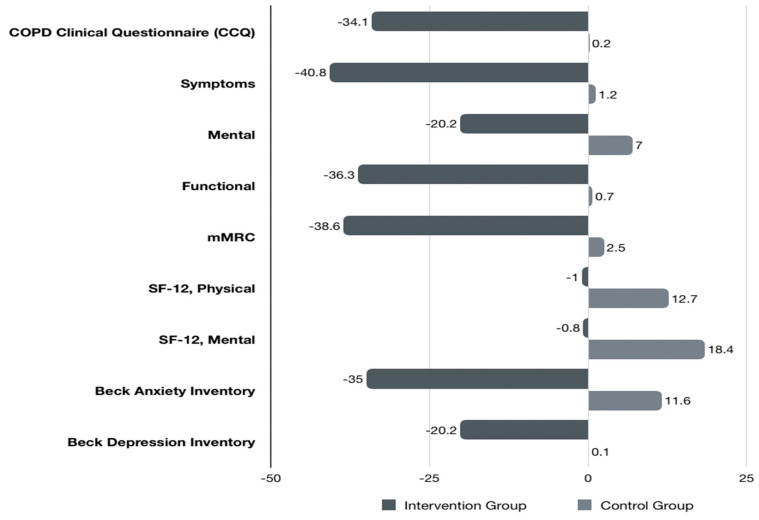
Percentage change score in BAI, mMRC, CCQ, BDI, and SF-12 in the two groups before and after the end of the follow-up period.

Questionnaires’ scores at baseline and after the 6-month follow-up period are shown in Table 3, Table 4 and Table 5, with significant improvements in all PROMs for the intervention group, while there was also a statistically significant difference compared to the control group receiving standard care. More specifically, the BAI score decreased significantly in the intervention group (−35%), whereas it increased by 11.6% in the control group (*p* < 0.001). Regarding the BDI scale, the control group did not show any change (0.1%), whereas the intervention group improved by −20.2% (*p* < 0.001) (Figure 2). The total score on the COPD assessment questionnaire decreased by −34.1%, compared to a +2% increase in the control group (*p* < 0.001). The same applies to all the subscales of this questionnaire. The Quality of Life Scale (SF12) subscale scores improved significantly in the intervention group, compared to the control group. Specifically, both the “Physical Health” and the “Mental Health” subscales increased by 12.7% (*p* < 0.001) and 18.4% (*p* < 0.05), respectively, in the intervention group, whereas a negative change was noted in the control group (−1% and −0.8% respectively, *p* > 0.05).

**Figure 3:**
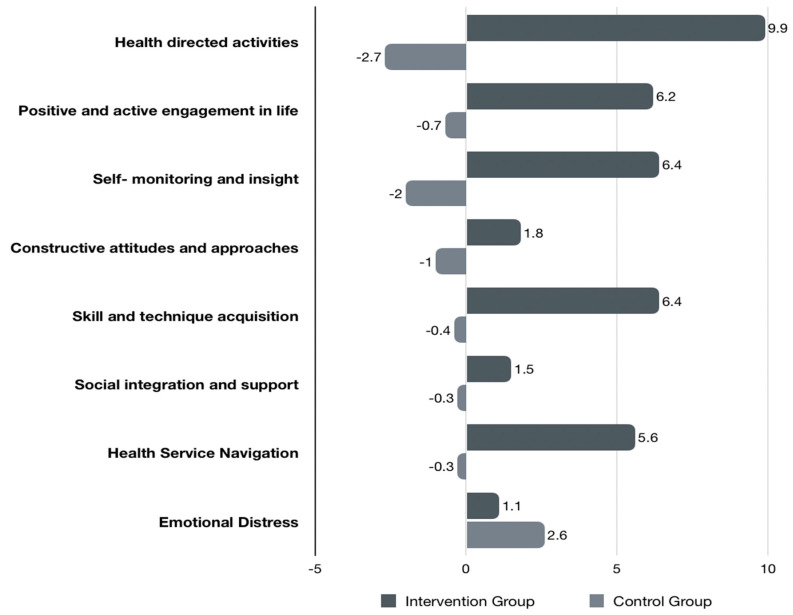
Percentage change score in HeiQ subscales in the two groups before and after the end of the follow-up period.

A significantly higher score for most of the subscales of the Health Education Impact Questionnaire was also noted in the intervention group (Table 4) reflected as an improvement in the subscales of “Browsing the Health Services” (intervention: + 5.6% vs. control: −0.3%), “Skill and technique acquisition” (intervention: +6.4% vs. control: −0.4%), “Self-monitoring and insight” (intervention: +6.4% vs. control: −2%), “Positive and active engagement in life” (intervention: +6.2% vs. control: −0.7%), and finally “Health “directed activities”(intervention: +9.9% vs. control: −2.7%) (Figure 3).

**Figure 4:**
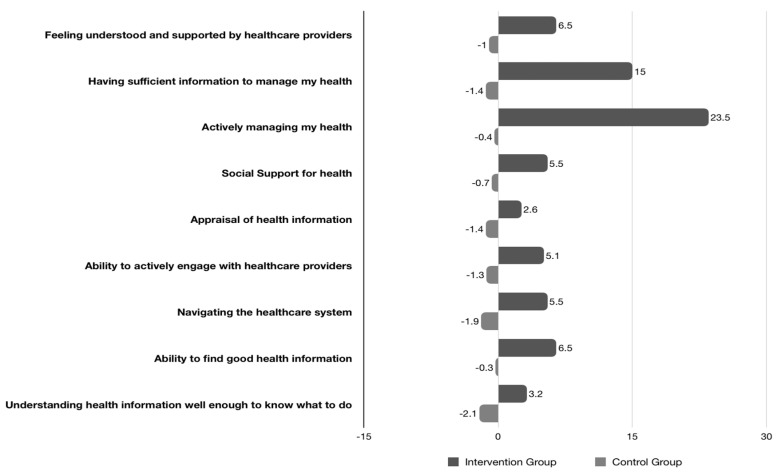
Percentage change score in HLQ subscales in the two groups before and after the end of the follow-up period.

When health literacy assessed by the Health Literacy Questionnaire (HLQ) scale was compared between baseline and at the end of the follow up, it was observed that most of the subscales showed a greater improvement in the intensive group (Table 5). More specifically, “Social Support for Health” had a +5.5% increase in the intervention group compared to a −0.7% decrease in the control group (*p* < 0.05) (Figure 4). A change by +6.5% of the intervention and −1% of the control group was observed in the “Feeling of understanding and support from healthcare providers” (*p* < 0.05). In “Having sufficient information to manage my health” the intervention group had a +15% improvement over the control group at −1.4% (*p* < 0.05). Additionally, the largest change was observed in “Active health management” with the intervention group reaching 23.5% compared to the control group decrease by −0.4% (*p* < 0.05) (Table 5).

## 4. Discussion

This RCT assessed the impact of a self-management intervention on PROMs, including quality of life, functional status, patient health literacy, depression, and anxiety in patients with mild COPD in Greece’s primary healthcare facilities. The intervention demonstrated significant and clinically meaningful improvement in all PROMs in the intervention compared to the control group, independent of age, place of residence, age at diagnosis of COPD, and the number of exacerbations in the previous year even though participants were enrolled in the study for a period of six months.

This is the first study to present findings from the implementation of a self-management program for patients with COPD in primary care in Greece. Interestingly, it seems that, in Greece, COPD self-management is underutilized, despite current national recommendations [38] proposing a holistic approach tailored to the needs of the Greek community and previous research showing the advantages of these interventions [39]. Indeed, a previous study in Greece found low adherence to recommendations [40], indicating that non-pharmacological management was not regarded as an essential component of care. Furthermore, another country-specific challenge could be the lack of effective communication and educational skills from healthcare professionals to support self-management in COPD or promote behavior change in these patients [41]. Therefore, this study could increase awareness of the importance of self-management within the Greek primary care community [41]. The educational component of the program was based on evidence-based best practices and the program was specifically designed to meet the specific needs of patients in a primary care setting. Our study’s intervention group also had frequent contact with one health professional, which appeared advantageous to the participants’ mental wellbeing, in accordance with previous studies [42].

Due to the heterogeneity of the patient-with-COPD population in terms of clinical presentation, disease severity, and rate of disease progression, and to demonstrate the efficacy of the self-management intervention, it appeared useful to include various PROM instruments. The inclusion of patients’ perspectives through PROMs in clinical trials has gained significance recently for a better understanding of disease impact on health-related quality of life [43]. Anxiety and depression, measured by the BAI and BDI, respectively, as well as the mental component of the CCQ, were all improved in participants assigned to the self-management intervention. A “dealing with breathlessness” component may have contributed to the improved effects on anxiety and depression; indeed, considering the “Thinking” negative cycle in the Breathing–Thinking–Functioning (BTF) model, depression and anxiety seem to affect and to be affected by breathing and also physical activity [44]. However, the baseline levels of anxiety and depression and the mental component of the CCQ in the intervention group were higher compared to usual care, resulting in room for improvement for this parameter. Nevertheless, a previous meta-analysis including studies conducted in primary care settings on COPD self-management outcomes also provided strong evidence for a meaningful improvement in mental health status in these patients [15]. In support of this, a recent meta-analysis assessing the effect of self-management interventions delivered in different care settings also showed better mean Hospital Anxiety and Depression Scale (HADS) scores for the self-management intervention compared to usual care [9].

Regarding health-related quality of life, the SF-12 physical and mental domain scores in the intervention group also showed significant improvements, but contradictory results were observed in prior studies using the same quality-of-life instrument following 3- to 12-month follow-up programs [45,46,47]. Moreover, we found clinically relevant improvements in a COPD-specific quality-of-life measure, the CCQ, among participants in the COPD self-management intervention group compared to the group receiving usual care. This finding aligns with a previous study that employed the same quality-of-life metric [48,49] and a recent review that indicates improvements in different metrics of health-related quality of life [48,49]. On the other hand, a significant improvement in dyspnea scores measured by the mMRC was found, in contrast to previous studies [50,51,52,53,54,55,56]. Nevertheless, mMRC scores indicated that, overall, participants had milder disease and were less symptomatic compared to a typical population in secondary and tertiary care.

Using the “Health Education Impact Questionnaire” (HeiQ) to evaluate patient education and self-management interventions, positive changes were observed in the interventional group for most of the domains of the HeiQ, in line with previous studies [14,57,58,59] showing improvement on some self-management-related domains. The training of the intervention and the regular follow up may have resulted in an increase in most of the domains of HeiQ (self-monitoring and insight). However, the HEIQ subscales of constructive attitudes and approaches, social integration and support, and emotional distress were not improved. This may be explained by limited in-person group-based interventions due to the COVID-19 pandemic. In addition, the HLQ demonstrated an overall improvement in the majority of its domains, as supported by a previous study [57]. Nonetheless, subscales regarding the appraisal and understanding of health information did not exhibit the same level of improvement, possibly influenced by the educational backgrounds of the participants.

Our findings contribute to the international literature by introducing a self-management program for patients with COPD, with positive effects in all PROMs assessed. Patients were educated in self-management techniques and were provided with the necessary tools to manage their condition effectively. Furthermore, our study highlighted the importance of communication and encouragement that a health professional could provide in self-management programs during the COVID-19 pandemic. It seems that our self-management intervention addressing mental health concerns was more effective than those focusing solely on symptom management. However, it is difficult to compare our findings with those of previous studies, as these often involve patients with varying degrees of COPD severity, different settings, and distinct characteristics of each self-management intervention.

### Strengths and Limitations

The present study had a number of advantages. First, it included the randomized and blinded study design, 6-month time frame, and examination of quality of life, functional status, patient health literacy, depression, and anxiety using validated questionnaires. Second, this study was conducted in a primary care setting, which is where the majority of patients with COPD receive their care with the potential to reach a large number of patients. On the other hand, the study had some limitations. Since it was a real-life implementation of a self-management program, the generalizability of the results is limited to patients who demonstrate a willingness to engage in comparable programs, increasing the risk of selection bias. It is also important to note that only individuals residing in urban areas were included in the study, with no representation from rural areas. Consequently, this could influence the level of adherence to instructions and potentially lead to more positive outcomes for patients in urban areas. Furthermore, this research was conducted during the initial phase of the COVID-19 pandemic, leading to a small sample size and limited in-person group-based interventions. Aside from that, a 6-month period was insufficient to determine whether the intervention had any effect on preventing future exacerbations, reducing hospitalizations, or sustaining the positive effect on PROMs. Lastly, in our study we included patients classified as GOLD Group A and B, as these patients tend to visit primary care more often. Moreover, we excluded patients with moderate-to-severe COPD labeled as Group C and D because they could potentially benefit more from these interventions, and we wanted to focus on “usual” patients in primary care. Future large-scale studies therefore may need to consider these factors.

## 5. Conclusions

In conclusion, our results suggest that a self-management intervention in COPD patients is an effective strategy for improving quality of life, functional and mental health status, and patient literacy about their disease in a primary care setting. The intervention program in Greece was effective in educating patients about self-management techniques and providing them with the necessary tools to manage their condition effectively. Future research could focus on the long-term impact of self-management interventions on the quality of life of patients with COPD, as well as the scalability and sustainability of these interventions for the less severe primary care population. Moreover, policymakers could utilize our findings to design and implement better self-management interventions in patients with COPD, and thus improve their overall quality of life.

## Figures and Tables

**Table 1:** Patients’ characteristics at baseline assessment.

	Total (*n* = 120)	Control Group (*n* = 80)	Intervention Group (*n* = 40)	*p*-Value
Gender, males	74 (62%)	53 (66%)	21 (53%)	0.14
Age groups				
<45	5 (4%)	4 (5%)	1 (3%)	0.05
46–55	13 (11%)	9 (11%)	4 (10%)	
56–65	37 (31%)	18 (23%)	19 (47%)	
>66	65 (54%)	49 (61%)	16 (40%)	
BMI > 25	95 (79%)	63 (79%)	32 (80%)	0.87
Smoking status				
Never/former	63 (53%)	42 (52%)	21 (52%)	0.99
Current	57 (47%)	38 (48%)	19 (48%)	
Marital status				
Married/in relationship	87 (73%)	58 (72%)	29 (72%)	0.99
Single	33 (27%)	22 (28%)	11 (28%)	
Level of education				
Primary level	66 (55%)	44 (55%)	22 (55%)	0.46
Secondary level	44 (37%)	31 (39%)	13 (32%)	
Tertiary level	10 (8%)	5 (6%)	5 (13%)	
Occupational status				
Not working *	37 (31%)	12 (15%)	6 (15%)	0.84
Working	18 (15%)	26 (32%)	11 (27%)	
Retired	65 (54%)	42 (53%)	23 (58%)	
Place of living				
Urban	67 (56%)	27 (34%)	40 (100%)	<0.001
Rural	53 (44%)	53 (66%)	0 (%)	
Comorbidities				
Arterial hypertension	58 (48%)	36 (45%)	22 (55%)	0.30
Cardiovascular disease **	44 (37%)	25 (31%)	19 (48%)	0.08
Diabetes type 2	27 (23%)	17 (21%)	10 (25%)	0.64
Depression	26 (22%)	16 (20%)	10 (25%)	0.53
Cancer	6 (5%)	5 (6%)	1 (3%)	0.37

* Unemployed, students, housekeeper. ** Coronary heart disease, heart failure, angina.

**Table 2:** Baseline questionnaire scores between the two groups.

Patient-Reported Outcome Measures	Total Population	Control Group	Intervention Group
	*n* = 120	*n* = 80	*n* = 40
Clinical COPD Questionnaire (CCQ)	1.7 ± 1.2	1.5 ± 1.2	2.1 ± 1.1 *
Symptoms	1.8 ± 1.2	1.7 ± 1.3	1.9 ± 1.0
Mental	1.6 ± 1.7	1.1 ± 1.4	2.4 ± 1.9 *
Functional	1.8 ± 1.6	1.5 ± 1.6	2.2 ± 1.5 *
mMRC	1.5 ± 1.1	1.2 ± 0.9	2.2 ± 1.0 *
Short Form Health survey (SF-12)			
Physical	39.8 ± 10.3	43.0 ± 1.2	33.2 ± 1.84
Mental	46.4 ± 11.7	49.5 ± 1.4	40.1 ± 2.2
Beck Anxiety Inventory	10.8 ± 11.5	7.4 ± 10.4	17.5 ± 10.8 *
Beck Depression Inventory	12.6 ± 10.4	10.3 ± 9.9	17.4 ± 9.9 *
Health Education Impact Scale			
Health-directed activities	2.6 ± 0.6	2.6 ± 0.1	2.4 ± 0.1
Positive and active engagement in life	2.8 ± 0.4	2.9 ± 0.1	2.6 ± 0.1
Self-monitoring and insight	2.8 ± 0.4	2.9 ± 0.1	2.7 ± 0.1
Constructive attitudes and approaches	2.9 ± 0.4	2.9 ± 2.7	2.7 ± 0.1
Skill and technique acquisition	2.7 ± 0.5	2.8 ± 0.1	2.3 ± 0.1
Social integration and support	2.9 ± 0.4	3.0 ± 0.1	2.7 ± 0.1
Health service navigation	2.9 ± 0.5	3.0 ± 0.1	2.7 ± 0.1
Emotional distress	2.9 ± 0.5	2.3 ± 0.1	2.7 ± 0.1
Health Literacy Questionnaire (HLQ)			
Feeling understood and supported by healthcare providers	3.0 ± 0.4	3.0 ± 0.1	2.9 ± 0.1
Having sufficient information to manage my health	2.8 ± 0.5	2.9 ± 0.1	2.5 ± 0.1
Actively managing my health	2.5 ± 0.5	2.7 ± 0.1	2.1 ± 0.1
Social support for health	2.9 ± 0.4	2.9 ± 0.1	2.7 ± 0.1
Appraisal of health information	2.7 ± 0.5	2.8 ± 0.1	2.5 ± 0.1
Ability to actively engage with healthcare providers	3.7 ± 0.6	3.8 ± 0.1	3.5 ± 0.1
Navigating the healthcare system	3.4 ± 0.7	3.7 ± 0.1	2.9 ± 0.1
Ability to find good health information	3.2 ± 0.8	3.4 ± 2.8	2.8 ± 0.1
Understanding health information well enough to know what to do	3.4 ± 0.8	3.6 ± 0.1	3.0 ± 0.2

* *p*-value < 0.05.

**Table 3:** Clinical COPD Questionnaire (CCQ), mMRC, SF-12, Beck Anxiety Inventory (BAI), and Beck Depression Inventory (BDI) questionnaire scores before and at the end of the follow-up period in control and intervention groups.

	Control Group		Intervention Group		
Questionnaires	Baseline	Follow Up	Baseline	Follow Up	*p*-Value ^#^
Clinical COPD Questionnaire (CCQ)	1.5 ± 1.2	1.5 ± 1.2	2.1 ± 1.2	1.4 ± 1.9 *	<0.001
Symptoms	1.7 ± 1.3	1.7 ± 1.3	1.9 ± 1.0	1.1 ± 0.8 *	<0.001
Mental	1.1 ± 1.4	1.2 ± 1.4	2.4 ± 1.9	1.9 ± 1.5 *	0.04
Functional	1.5 ± 1.6	1.5 ± 1.6	2.2 ± 1.5	1.4 ± 1.3 *	<0.001
mMRC	1.2 ± 0.9	1.2 ± 0.9	2.2 ± 1.0	1.4 ± 1.03 *	<0.001
SF-12					
Physical	43.0 ± 1.2	42.7 ± 1.9	33.2 ± 1.8	39.3 ± 1.9 *	<0.001
Mental	49.5 ± 1.4	49.0 ± 1.4	40.1 ± 2.2	45.2 ± 1.9 *	0.003
Beck Anxiety Inventory	7.4 ± 10.4	8.3 ± 9.9 *	17.5 ± 10.8	11.4 ± 9.9 *	<0.001
Beck Depression Inventory	10.3 ± 9.9	10.3 ± 9.9	17.4 ± 9.9	13.9 ± 9.7 *	<0.001

* Paired test’s *p*-value < 0.05, ^#^ generalized linear models adjusted for age, place of residence, age at diagnosis of COPD, and the number of exacerbations in 2019.

**Table 4:** Health Education Impact Questionnaire subscale scores before and at the end of the follow-up period in control and intervention groups.

	Control Group		Intervention Group		
Questionnaires	Baseline	Follow Up	Baseline	Follow Up	*p*-Value ^#^
Health Education Impact Scale					
Health-directed activities	2.6 ± 0.2	2.6 ± 0.1	2.4 ± 0.1	2.7 ± 0.1 *	0.007
Positive and active engagement in life	2.9 ± 0.1	2.9 ± 0.1	2.6 ± 0.1	2.8 ± 0.1 *	0.004
Self-monitoring and insight	2.9 ± 0.1	2.9 ± 0	2.7 ± 0.1	2.8 ± 01 *	0.001
Constructive attitudes and approaches	3.0 ± 0.1	2.9 ± 0.1	2.7 ± 0.1	2.8 ± 0.1	0.24
Skill and technique acquisition	2.8 ± 0.1	2.8 ± 0.1	2.3 ± 0.1	2.5 ± 0.1 *	0.03
Social integration and support	3.0 ± 0.1	3.0 ± 0.1	2.7 ± 0.1	2.7 ± 0.1	0.48
Health service navigation	3.0 ± 0.0	3.0 ± 0.1	2.7 ± 0.1	2.8 ± 0.1 *	0.04
Emotional distress	2.3 ± 0.1	22.4 ± 0.1	2.7 ± 0.1	2.8 ± 0.1	0.80

* Paired test’s *p*-value < 0.05, ^#^ generalized linear models adjusted for age, place of residence, age at diagnosis of COPD, and the number of exacerbations in 2019.

**Table 5:** Health Literacy Questionnaire (HLQ) subscale scores before and at the end of the follow-up period in control and intervention groups.

	Control Group		Intervention Group		
Questionnaires	Baseline	Follow Up	Baseline	Follow Up	*p*-Value ^#^
Health Literacy Questionnaire (HLQ)					
Feeling understood and supported by healthcare providers	3.0 ± 0.1	2.9 ± 0.1	2.9 ± 0.1	3.1 ± 0.1 *	0.002
Having sufficient information to manage my health	2.9 ± 0.1	2.9 ± 0.1	2.6 ± 0.1	2.9 ± 0.1 *	<0.001
Actively managing my health	2.7 ± 0.1	2.7 ± 0.1	2.1 ± 0.1	2.6 ± 0.1 *	0.001
Social support for health	3.0 ± 0.1	3.0 ± 0.1	2.7 ± 0.1	2.9 ± 0.1 *	0.001
Appraisal of health information	2.8 ± 0.1	2.8 ± 0.1	2.5 ± 0.1	2.6 ± 0.1	0.09
Ability to actively engage with healthcare providers	3.8 ± 0.1	3.8 ± 0.1	3.5 ± 0.1	3.7 ± 0.1 *	0.01
Navigating the healthcare system	3.7 ± 0.1	3.0 ± 0.1	2.9 ± 0.1	3.1 ± 0.1 *	0.03
Ability to find good health information	3.4 ± 0.1	3.4 ± 0.1	2.7 ± 0.1	2.9 ± 0.1 *	0.04
Understanding health information well enough to know what to do	3.6 ± 0.1	3.6 ± 0.1	3.0 ± 0.2	3.1 ± 0.2	0.12

* Paired test’s *p*-value < 0.05, ^#^ generalized linear models adjusted for age, place of residence, age at diagnosis of COPD, and the number of exacerbations in 2019.

## Data Availability

The data presented in this study are available on request from the corresponding author. The data are not publicly available due to privacy restrictions.

## Supplementary Materials

The following supporting information can be downloaded at: https://www.mdpi.com/article/10.3390/medicina60030377/s1.
