# Supplementary material for: A Chronic Obstructive Pulmonary Disease Self-Management Intervention for Improving Patient-Reported Outcomes in Primary Care in Greece
Source: Medicina (Kaunas). 2024 Feb 23;60(3):377. doi: 10.3390/medicina60030377 (PMC10972103; doi:10.3390/medicina60030377)
Supplement: Supplementary file 1 [file medicina-60-00377-s001.zip › medicina-2866850-supplementary.pdf]

A. Control group.

***Phase 1*:**

- The interviews were conducted by family doctors working in primary healthcare facilities. Individuals/patients were briefed on the subject and purpose of the research, and the importance of their participation.
- Those who agreed to participate signed a consent form and were assured that all information would be confidential.
- Subsequently, participants completed a questionnaire regarding gender, age, occupation, educational level, marital status, smoking, etc.
- The remaining questionnaires were distributed to 80 participants.
- Questionnaire completion period was from February to June 2020.

***Phase* 2:**

- Due to the COVID-19 Pandemic, the first author communicated with the participants via telephone and completed the questionnaires for the second/final time.
- Second period for completion of the questionnaires was from July to August 2020.

B. Intervention Group.

Phase 1: Each participant was briefed on the objective of the study, their voluntary participation, and signed a consent form.

- Demographic data were collected, and the remaining questionnaires were collected.
- A "training book" was given **only** to the intervention group, which was created for the study.
- The questionnaires were completed at an individual level (participant-researcher) in a space separated from the other clinics of the structure.
- Participants were presented with a PowerPoint summary of the "Training Book" with simultaneous active discussion.
- Participants were introduced to breathing games and strengthening exercises and were advised to also use them at home.

***Phase 2*:** This phase was comprised of **five sessions**, which occurred approximately once a month for five consecutive months. In each session, the following subjects were presented and explained: 1. Pathophysiology of COPD, 2. Risk Factors, 3. Symptom recognition, 4. Staging of the Disease and Recognition of the stage to which the individual belongs, 5. Exacerbation recognition training, 6. Exacerbation prevention training, 7. Training with Breathing strengthening exercises and muscle strengthening exercises, and 8. Proper Use of Inhalers.

- ***1st Session*** *- March 2020*: A telephone communication (due to COVID-19 measures) was made, in which the researcher (first author) explained how to read the "Training Book" to the participants.
- ***2nd Session***: A second telephone session (due to COVID-19 measures) was held about a week after the first, where the researcher (first author) answered any questions about the "Training Book".
- ***3rd Session****: The researcher (first author) held individual sessions in person, with a difference of approximately one month from the 1st session.*
- ***4th Session***: Individual training continued, with one week difference from the 3rd Session and included: Repetition of previous training chapters and Vaccination information for COPD patients
- ***5th Session***: The dates were approximately three months from the 1st Telephone Session. Individual training continued with 1. Repetition of previous training chapters 2. information on the legal rights of people with COPD and disability; and 3. Exacerbation of COPD and seasonal allergies, 4. training in dealing with exacerbations of seasonal allergies; and 5. Strategic plan "Ability to Fly" for people with COPD, and 6. Truths and Myths of COPD.

***Phase 3*:** The control subjects were asked, one month after (July-August) the 5th training session, to complete the questionnaires a second time.
